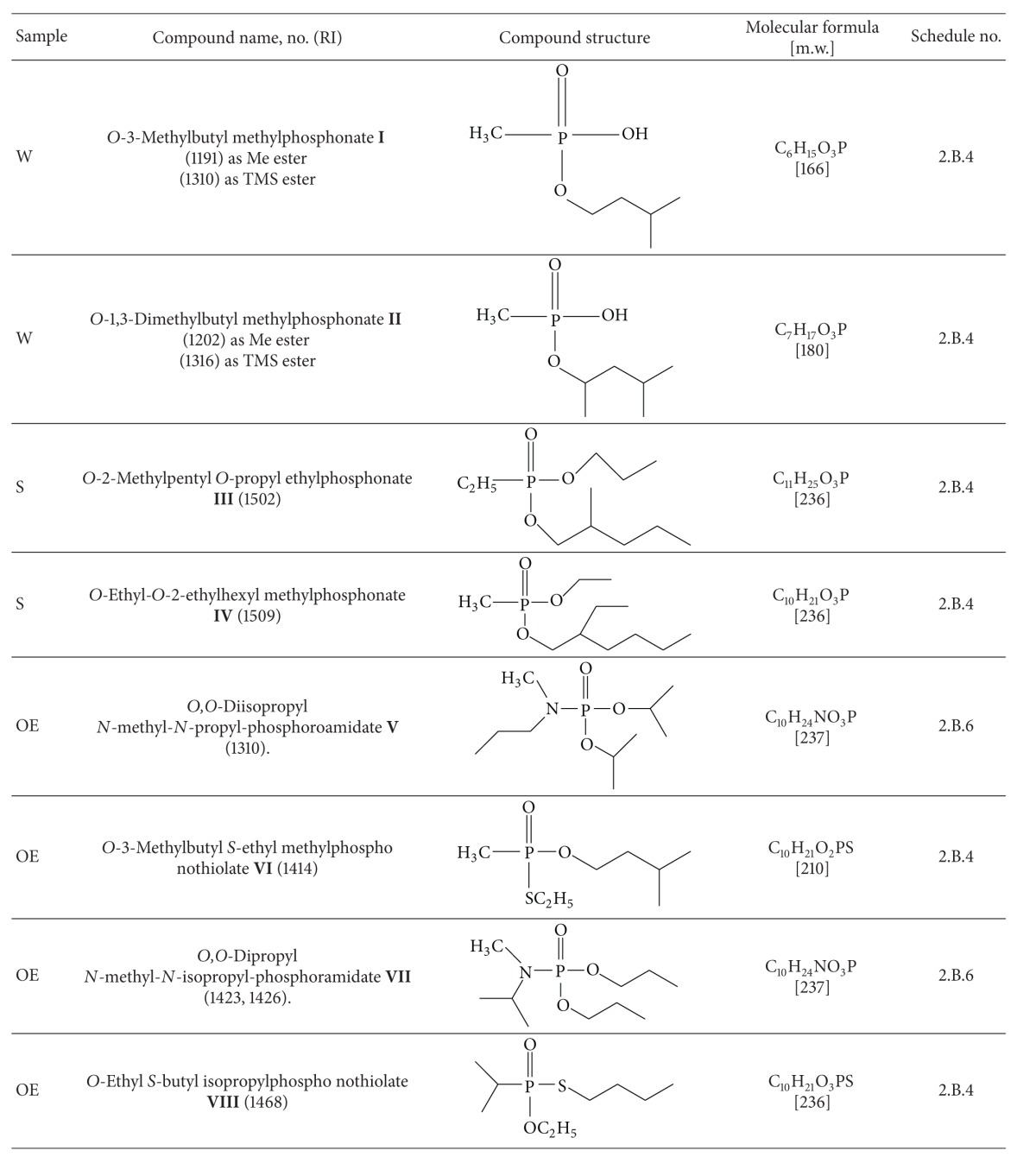# OPCW Proficiency Test: A Practical Approach Also for Interlaboratory Test on Detection and Identification of Pesticides in Environmental Matrices

**DOI:** 10.1155/2014/542357

**Published:** 2014-01-22

**Authors:** Leszek Konopski, Pingfeng Liu, Wuri Wuryani, Maciej Śliwakowski

**Affiliations:** ^1^OPCW Inspectorate, Inspection Management Branch, Johan de Wittlaan 32, 2517 JR The Hague, The Netherlands; ^2^Institute of Industrial Organic Chemistry, Annopol 6, 03-236 Warsaw, Poland; ^3^OPCW Laboratory, Technical Support Branch, Heulweg 28-30, 2288 GN Rijswijk, The Netherlands

## Abstract

An overview of general strategy, standard procedures, and critical points, which may be found during carrying out an OPCW Proficiency Test concerning detection and identification of scheduled compounds relevant to Chemical Weapon Convention, has been presented. The observations have been illustrated following the case of the Eight OPCW Designated Laboratories Proficiency Test, which was performed in the OPCW Laboratory in Rijswijk in November and December 2000. Various useful hints, comments, and practical observations concerning the case study have been included as well. The same methodology and procedures may be also applied for detection, identification, and environmental analyses of pesticides and biocides, especially organophosphorus compounds.

## 1. Introduction

Proficiency test (commonly called also *Round Robin* test) is an interlaboratory test, which proves that participating laboratories are able to perform their work in a manner, which is generally believed to be correct in the scope of analyzed compounds. It is recommended that the laboratories developed an internationally recognized quality system to prove their reliability.

The aim to participate in the Organization for the Prohibition of Chemical Weapons (OPCW) Proficiency Test is that the Member States laboratories seek to become a designated laboratory for analyses largely connected with the CWC. In practice, the environmental analyses for the presence of scheduled compounds and their metabolites are mainly performed. The laboratories which regularly participate and perform successfully proficiency test may be then certified as OPCW Designated Laboratories. The condition is to accomplish successfully proficiency test for at lest three times consecutive and to obtain accreditation by an internationally recognized accreditation body for tasks for which they are seeking designation [1].

Only a designated laboratory may be taken into account to perform offsite analyses of any CWC related samples taken offsite during inspection activities, usually Challenge Inspection (CI) or Alleged Use Investigation AUI. Although no such a case had taken place up to now, a possibility to participate in offsite analyses is challenging for many leading laboratories of CWC Member States. Usually, at least twenty laboratories participated in every proficiency test (performed twice a year from 1997).

Samples are prepared by a designated laboratory selected among participants and simultaneously sent to the remaining laboratories. Usually, three environmental samples are prepared, generally soil, water, wipe, or organic wipe extract, but sometimes also nonsoil solid samples, as used gaskets or paintings. The samples are spiked with scheduled compounds; between 4 and 9 spiking chemicals are present in all samples altogether. The contents of scheduled compounds are unknown but not less than 1–10 mg/kg (i.e., ppm *m/m*). Blank samples of appropriate matrices are also supplied together with the real ones. The test results must fulfill the following acceptance criteria [2, 3].

The results should be supplied to OPCW Laboratory during 15 calendar days after receiving. The analysis reports should contain not only the results (sample chemical name, structural formula, CAS number, if available, and appropriate structural information if necessary to identify the chemicals), but also the convincing evidences supporting the obtained findings (comparison with data of standards, data from analytical databases, and/or interpretation of spectra), sample preparation details, chromatograms, spectra, and so forth. At least two different analysis techniques, preferably spectrometric (El GC/MS, CI GC/MS, LC/MS, (GC)/IR, (GC)/NMR in ^1^H, ^13^C, ^31^P, or ^19^F mode, etc.) should give consistent results. Only chemicals relevant to the test aims should be reported, that is, the spiking chemicals and/or their degradation products. The acceptance criteria give positive points for every correct identification. False positive results (artifacts) must not occur, and reporting of such a chemical will constitute failure of the test. Thus, among possible decomposition products, only chemicals which can be formed in sample matrix may be reported. False negative results arising from not finding a spiking chemicals or its degradation products are scored negatively (−1 point for every chemical).

In case of organophosphorus compounds, only the side chain of the P–C bond must be fully identified. The specific identification of the location of alkyl groups in the *O*-alkyl or *O*-cycloalkyl side chains is not required. However, the total number of carbon atoms in every group must be reported correctly. Identification of minor constituents (as impurities) of spiking chemicals is not required, and reporting them is not considered incorrect. However, it will not be added to the score. Only qualitative results have to be reported.

The test reports are then sent by OPCW Lab to another designated laboratory for evaluation, since OPCW Laboratory participates in proficiency test basically as an organizing and coordinating unit. However, the Lab actively cooperates in evaluation of results and in acceptance of the performance criteria. The OPCW Laboratory activities are covered by the Quality System [4]. In December 2000, the Lab passed successfully external audit by *Raad van Akreditatie* (the Dutch accreditation body) and obtained accreditation actualized every 3 years.

Although the OPCW Laboratory is not officially engaged in the proficiency tests as a regular participant, the same analytical work as in participating laboratories has been carried out also in OPCW Laboratory in the same time frames. The reason is to better understand the possible critical points of analysis and to better perform the evaluation duties. Only routine equipment available in the Lab (El and CI GC/MS, and GC/NPD and FPD) was currently used.

The Eight OPCW Proficiency Test was scheduled to be performed in November 2000. Since the Lab senior analytical chemist, which has usually been involved in the proficiency test analyses was present during the samples spiking and preparation in a designated laboratory, his direct participation in the proficiency test performing was not recommended for obvious reasons. That is why only two AC inspectors and a Laboratory technician were this time directly involved in the performance test.

## 2. Materials and Methods

### 2.1. Sample Preparation

Three samples received in the Laboratory on 10 November were marked as follows: water sample (W), soil sample (S), and organic extract from a wipe (OE). The blank samples enclosed were marked WB, SB, and OEB, appropriately.

In sample preparation, all the samples were treated in a manner described in the corresponding Work Instructions [5–7] with some minor changes.

1. Very often, the matrix contains important amounts of additives. Thus, the soil was heavily contaminated with fuel oil, the water sample with polyethylene glycols, and the organic extract contained considerable amounts of different organic compounds. Thus, after a preliminary routine treatment and concluding that matrix may interfere in spiking chemicals identification, the samples were precleaned.
  - The dichloromethane extract from soil sample was then purified on short silica gel column (prewashed with hexane), and most of matrix fuel—hydrocarbons was eliminated by washing the column with dichloromethane and subsequently with acetone. The relevant to the Chemical Weapon Convention scheduled chemicals with medium polarity (as phosphonic acid diesters) were found mainly in acetone fraction, which was almost free of hydrocarbons. Alternatively, the dichloromethane extract from soil sample was washed on silica column, first with dichloromethane (to remove hydrocarbons) and then with acetone.
  - The dichloromethane extract from water was derivatized and then subsequently washed with water. In such a way, the polyethylene glycols were reextracted to the water phase.
2. In the silylation procedure, dichloromethane was first added to the evaporated samples before silylation, and the solid residue from the tube walls was scratched with a glass rod and sonificated on an ultrasonic bath to enable solubilizing the sample. The trimethylsilyl (TMS) derivatives were performed with BSTFA in acetonitrile instead of THF in the same concentration (1 : 1 *V/V*).
3. Additionally, parallel to the TMS derivatives, the methyl (Me) derivatives were obtained with diazomethane solution obtained in standard method from Diazald (*N*-nitroso-*p*-toluenesulfonamide) and potassium hydroxide in ethyl ether and subsequent distillation. The methylation with diazomethane/ether solution was carried out in methanol at room temperature for approximately 5 minutes, and the excess of diazomethane was then decomposed by the addition of a drop of 1% acetic acid in methanol.
4. The diazomethane derivatization was also performed, when the presence of free phosphonic acids in the treated material was suspected. Such a procedure allowed avoiding contamination of the injector and the column with a nonvolatile phosphonic acid, which could be there derivatized after every injection containing a silylating agent and then released. This could produce false positive identifications and/or mask presence of real spiked chemicals. To avoid the injector/column contamination with nonvolatile chemicals, the less possible numbre of maximally diluted samples was always injected.
5. The sample after such a “preventive” diazomethane derivatization had sometimes in the GC/MS chromatogram tendency to peak splitting and to form at front of some peaks one or more additional “forpeaks” with the same mass spectrum as the main peak. The reason of this phenomenon is not clear, but it may be due to the presence of excess of methanol, nondecomposed diazomethane and even traces of water. To eliminate these artifacts, the methanol solutions after derivatization were concentrated in nitrogen stream at 30°C, water (1 mL) was added, and the organic was reextracted with 1 mL of dichloromethane. Organic layer was dried with sodium sulfate and filtered.

### 2.2. Hydrolysis of Samples

The precleaned and concentrated dichloromethane extracts of samples were hydrolyzed, if necessary, to prove presence of suitable alkylphosphonic acids and appropriate alcohols.

The *O*-esters hydrolyzed already in 2 M sodium hydroxide at 30–40°C overnight, whereas *S*-alkyl thioesters required 5 M concentration, and *O,O*-dialkyl *N,N*-dialkylphosphoroamidates did no undergo hydrolysis in the above conditions.

The following general procedure was used. 

*General Procedure for Hydrolysis of Diesters (R*_*1*_ *O)P(=O)R(O(S)R*_*2*_ *).*


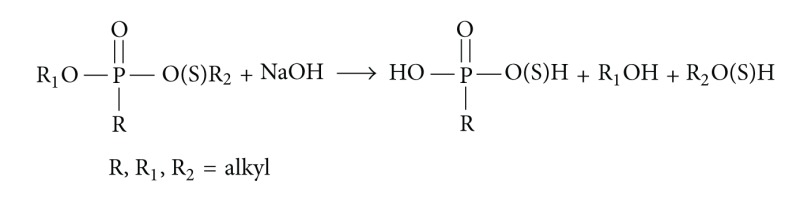

1. Less than 1 mg of diester and 0.5 mL of aqueous sodium hydroxide (2 M or 5 M solution, the last one should be used for *S*-alkyl thioesters) was heated overnight at 30–40°C.
2. Next day, the mixture was cooled down. 2 mL of H_2_O was added, the solution transferred to a 4 mL vial, extracted with (2 × 1 mL) CH_2_Cl_2_, and two phases were separated.
3. The obtained alcohols or thiols R_1_OH and R_2_(S)OH remained in the organic dichloromethane layer. The organic layer was dried and concentrated at RT and alcohols could be identified using GC/MS.
4. The water layer was neutralized to pH = 7 and passed through a preconditioned SCX cation exchange cartridge, free acids solutions were evaporated to dryness, and the derivatization was performed (methylation with diazomethane [2] and silylation with BSTFA).
5. Alternative way to process: the water fraction was to neutralize to pH = 7, evaporate to dryness, and add solution of HCl in dry MeOH to dissolve the dry residue. Then the solution was evaporated to dryness in a mild nitrogen flow in order to remove HCl. Next, add MeOH and treat the methanolic solution with CH_2_N_2_/ether. If a precipitate is formed during methylation, the solution was filtered or centrifuged, if necessary.
6. The hydrolysis procedure was done for the soil sample S: acetone fraction (after washing in hexane prewashed silica column with dichloromethane/acetone to remove the hydrocarbons matrix) was evaporated at 30°C/in mild nitrogen flow and treated as above with 2 M NaOH.
7. The same procedure was also carried out for organic extract sample OE, but hydrolysis was performed in 5 M NaOH overnight.

### 2.3. Preparation of Reference Substances

To confirm the identifications, certain amounts of reference substances have been synthesized. The same retention time/retention index, as well as identical mass spectra, is additional evidence for the identity of the found compound and the reference substance. All detected chemicals were thus synthesized to confirm their identity as follows:

- dialkyl alkylphosphonates,
- *O,O*-dialkyl *N,N*-dialkylphosphoroamidates,
- monoalkyl alkylphosphonates,
- dialkyl alkylphosphonothiolates.

*(i) General Procedure to Prepare Diesters and Mixed Diesters of Alkylphosphonic Acids.* R_1_O–P(=O)R–OR_2_.

*(ii) Dialkyl N,N-Dialkylphosphoroamidates.* R_1_O–P(=O)(NR_3_R4)–OR_2_


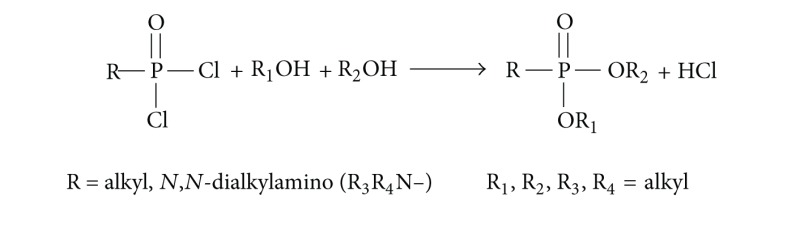


If the mixed diesters were synthesized, in addition to expected asymmetric diester R_1_O–P(=O)R–OR_2_, also both symmetrical ones R_1_O–P(=O)R–OR_1_ and R_2_O–P(=O)R–OR_2_ were always obtained as byproducts. Depending on the alcohol reactivities (MeOH ≫ EtOH > PrOH > i-PrOH > C_4_OH, etc.) their amounts should be controlled to assure a proper excess (from two-to even tenfold) of the less reactive starting alcohol. Since the reactivities of lower alcohols are very high, the dichloride must be always dissolved first in less reactive alcohol. The free acid, which may be present in dichloride or may be formed by its hydrolysis by the water present in alcohols (especially methanol and ethanol), must be at the end of treatment transformed in Me derivative with diazomethane (see (3) and (4) in Section 2.1).

1. Prepare mixture of alcohols R_1_OH and R_2_OH in a ratio depending on their reactivity.
2. Add approximately 10 mg (or 10 *μ*L) of appropriate alkylphosphonic dichloride (R = alkyl) or dialkylamidophosphoric dichloride (R = R_3_R_4_N–). Some drops of pyridine were in some cases added in order to trap HCl.
3. The reaction is done at 30°C~40°C overnight.
4. Next day, the reaction mixture was cooled down to RT. 10 *μ*L of the reaction mixture and 3–5 drops of acetone were dissolved in 1 mL of water. Shaken and washed with CH_2_Cl_2_ (2 × 0.5 mL), the organic extract was dried with Na_2_SO_4_ and then filtered. The resulting dichloromethane solution was evaporated almost to dryness at 30°C with a mild N_2_ flow. 1 mL of MeOH was added and methylated with excess of CH_2_N_2_ to avoid injection of free acids on GC/MS.

*(iii) General Procedure to Prepare Monoesters of Alkylphosphonic Acids.*


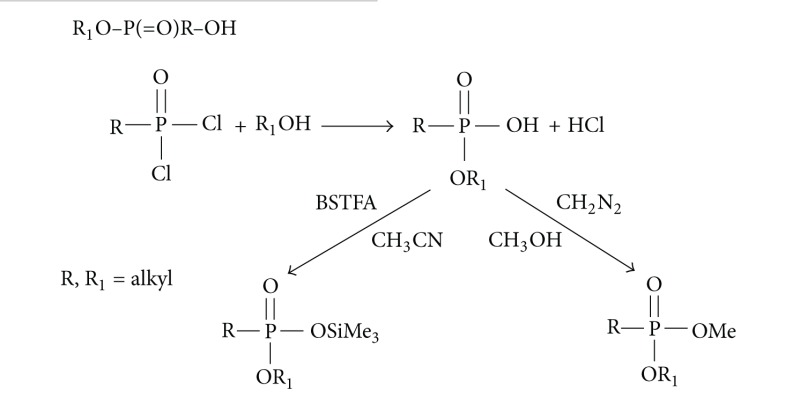

1. Prepare ca. 10 mg/mL solution of the appropriate dichloride R–P(=O)Cl_2_ in CH_2_Cl_2_.
2. Prepare monoequivalent solution of the appropriate alcohol R_1_OH in CH_2_Cl_2_ (1 : 1 mole/mole).
3. Add 0.5 mL of dichloride solution to 0.5 mL of the alcohol solution and shake them; the reaction was done at 30–40°C overnight.
4. Next day, cool down the solution, add 1 mL H_2_O, shake, and separate the two phases. Then, acidify the water phase to pH = 2. Free acids present in the water phase wash again with l mL of fresh CH_2_Cl_2_. The dichloromethane phase dry with sodium sulfate, and evaporate it on a rotary evaporator to dryness for preparation of methyl or TMS derivative, using etheral CH_2_N_2_/CH_3_OH or BSTFA/CH_3_CN, appropriately.

*(iv) General Procedures to Synthesize O,S-Dialkyl Alkylphosphonates*.


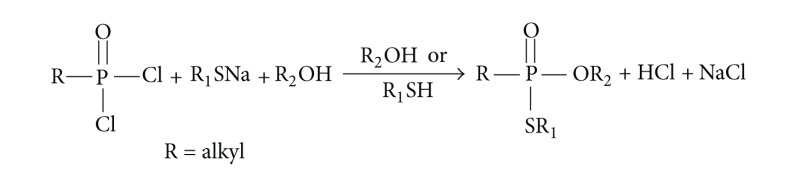


If the mixed *O,S*-thioesters have to be synthesized, the free thiol R_1_SH must be first transferred in its sodium salt R_1_SNa. Some of such salts are commercially available (method A). This thiol sodium salt is not much more reactive than alcohol R_2_OH, but because R_1_SNa is solid and insoluble in alcohol R_2_OH, the dichloride must be first dissolved in alcohol frozen to −20°C and then the thiol sodium salt has to be added as soon as possible. Moreover, this reaction is heterogenic and requires shaking at the beginning but is faster that synthesis of an ordinary *O,O*-diester.

Alternatively, if the thiol sodium salt is not commercially available, it may be prepared in situ from thiol and sodium (method B):

(1) $$\begin{array}{c} {\text{R}}_{1}\text{SH}+\text{Na}⟶{\text{R}}_{1}\text{SNa}+{\text{H}}_{2}↑ \end{array}$$

In such a case, the reaction is performed in thiol as a solvent, and the alcohol is added later on. This reaction is homogenic and also faster that ordinary diester synthesis.

In both cases, in addition to expected asymmetric diester R_1_S–P(=O)R–OR_2_, also both symmetrical ones: R_2_O–P(=O)R–OR_2_ (usually a main reaction product in method A, absent in method B) and R_1_S–P(=O)R–SR_1_ (absent in method A, usually a main reaction product in method B) were obtained as byproducts. That is why the amount of alcohol should be minimized only in method A, but in both methods, after dichloride addition, alcohol and thiol sodium salt must be mixed up as quickly as possible.

Syntheses of exemplary mixed *O,O*-diesters using both methods (A and B) were as follows. 

*Method A (S*-*Ethyl O-3-Methylbutyl Methylphosphonothiolate).* 18.8 mg of commercial solid sodium thiophenolate (EtSNa, 2 equivalents) was loaded into a 4 mL vial. In the other vial, 13.3 mg of MeP(O)Cl_2_ (1 equivalent) put and dissolved it as quickly in 0.4 mL of 3-methylbutyl alcohol and frozen to −20°C. Then, the dichloride solution in cold alcohol was added into the vial with EtSNa as quickly as possible. The vial was shaken for 10 seconds, then 0.5 mL CH_3_CN was added and the vial content was mixed for another 5 min and left it at 30–40°C for one hour.

10 *μ*L of the above reaction mixture was transferred into a 4 mL vial. 1 mL of water was added and the resulting liquid was washed with CH_2_Cl_2_ (3 × 1 mL). Organic phase dried over anhydrous MgSO_4_, and the solution was filtered and evaporated almost to dryness using a mild nitrogen flow at 30°C. Then 1 mL of CH_3_OH was added and methylated with CH_2_N_2_ in order to derivatize possible traces of free acids. Methanolic solution was evaporated almost to dryness using mild N_2_ flow at 40°C, l mL of water add and reextract with CH_2_Cl_2_. Organic extract after drying with MgSO_4_ and filtration is ready to be injected into GC/MS. The reaction mixture contained two main products: the expected *S*-ethyl-*O*-3-methylbutyl methylphosphonothiolate (35%) and *O,O*-bis(3-methylbutyl) methylphosphonate (65%, a byproduct but the major component).

*Method B (O-Ethyl S-Butyl Isopropylphosphonothiolate).* 5.7 mg of sodium (1 equiv.) was dissolved in 150 *μ*L of butanethiol, heating mildly to about 40°C in a 4 mL vial and leaving a space to evacuate hydrogen (do not screw the vial tightly). Then the reaction mixture was cooled down, 35 *μ*L of iPr–P(=O)Cl_2_ (1 equiv.) was added, and, as soon as possible, 20 *μ*L of EtOH. The resulting solution was kept at 30~40°C for one hour, and then treated in the same procedure as described for method A. The reaction mixture contained two main products: the expected *O*-ethyl-*S*-butyl isopropylphosphonothiolate (25%) and *S,S*-dibutyl isopropylphosphonodithiolate (75%, a byproduct), as well as dibutyl disulfide Bu_2_S_2_ (the major component).

### 2.4. Chromatographic Measurements

GC/NPD/FID was performed on HP 6890E gas chromatograph equipped with HP5 MS fused silica column, 25 m × 0.25 mm i.d., film thickness 0.25 *μ*m, in the following: program: 40°C (2 min)↗280°C (10°C/min), then 280°C for 10 min (total GC time: 36 min), carrier gas: helium, flow rate 1.3 mL/min. The chromatograph was equipped with both flame ionization detector (FID) and phosphorus nitrogen detector (NPD). All organic compounds were detected with the FID, but only compounds containing phosphorus and nitrogen were detected with the NPD detector. The GC conditions and column were arranged in such a manner that retention times (RT) were roughly the same as in GC/MS, and the FID chromatogram peaks intensities were approximately similar as in El GC/MS the total ion current (T1C).

GC/MS was performed on HP 6890E gas chromatograph coupled with a quadrupole mass detector HP 5972 MSD. Column: CPSil 5B, 30 m × 0.25 mm i.d., film thickness 0.25 *μ*m, in the following: program: 40°C (2 min)↗280°C (10°C/min), then 280°C for 10 min (total GC time: 36 min), carrier gas: helium, flow rate 0.9 mL/min. The ion source worked in both electron ionization (El) at 70 eV and chemical ionization (CI) modes. Methane and isobutane were used as reagent gases. selected ion monitoring (SIM) technique was used to show chromatograms of extracted ions at given *m/z* to confirm purity and identity of the studied chemicals. The extracted ion chromatograms were used together or alternatively to TlC.

Mass spectra (MS) were deconvoluted using HP and AMDIS programs, and different databases: NIST 97 library, OPCW Data Basis of Scheduled Compounds, and various user's databases containing scheduled compounds were used as well.

Additionally, the Kovats retention indices (RI or I) were calculated according to the formula below and compared with a RI database [9].

For temperature programmed chromatography, the Kovats index is given by [10]

(2) RI=100×[n−(N−n)tr(unknown)−tr(n)tr(N)−tr(n)],

where RI = Kovats retention index, *n* = the number of carbon atoms in the lower *n*-alkane, *N* = the number of carbon atoms in the higher *n*-alkane, and *t*_*r*_ = the retention time.

## 3. Results

The following scheduled chemicals were found in the samples, applying the appropriate Recommended Operation Procedures, Standard Operation Procedures, Work Instructions, and former OPCW Laboratory Reports discussed in detail in previous sections [3–7, 9–12] (Table 1).

The results were consistent with the list of spiking chemicals which were used in the Eight Official OPCW Proficiency Test. The detailed results of this test are presented in the OPCW Laboratory Report [11].

## 4. Discussion

**Figure 1:**
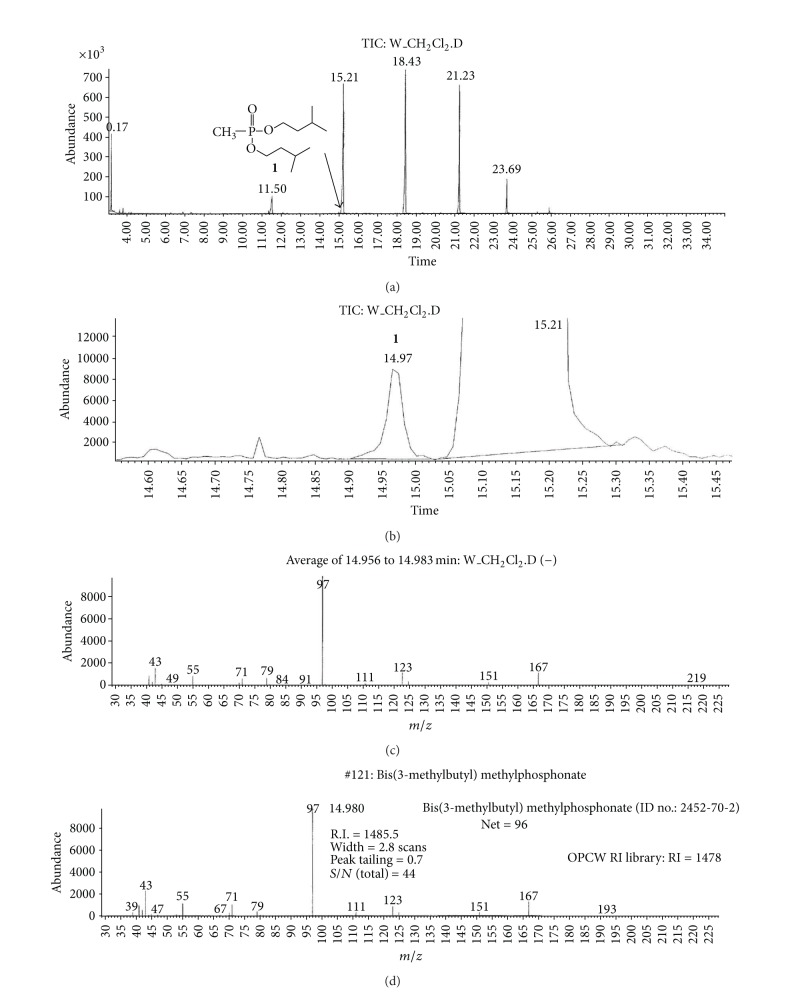
(a) and (b) TIC of raw dichloromethane extract from water sample W; (c) Mass spectrum of a minor component at *R*_*T*_ = 14.98 min, probably an impurity of one of analytes; (d) library search results—the studied compound was identified as bis(*O*-3-methylbutyl) methylphosphonate **1**.

**Figure 2:**
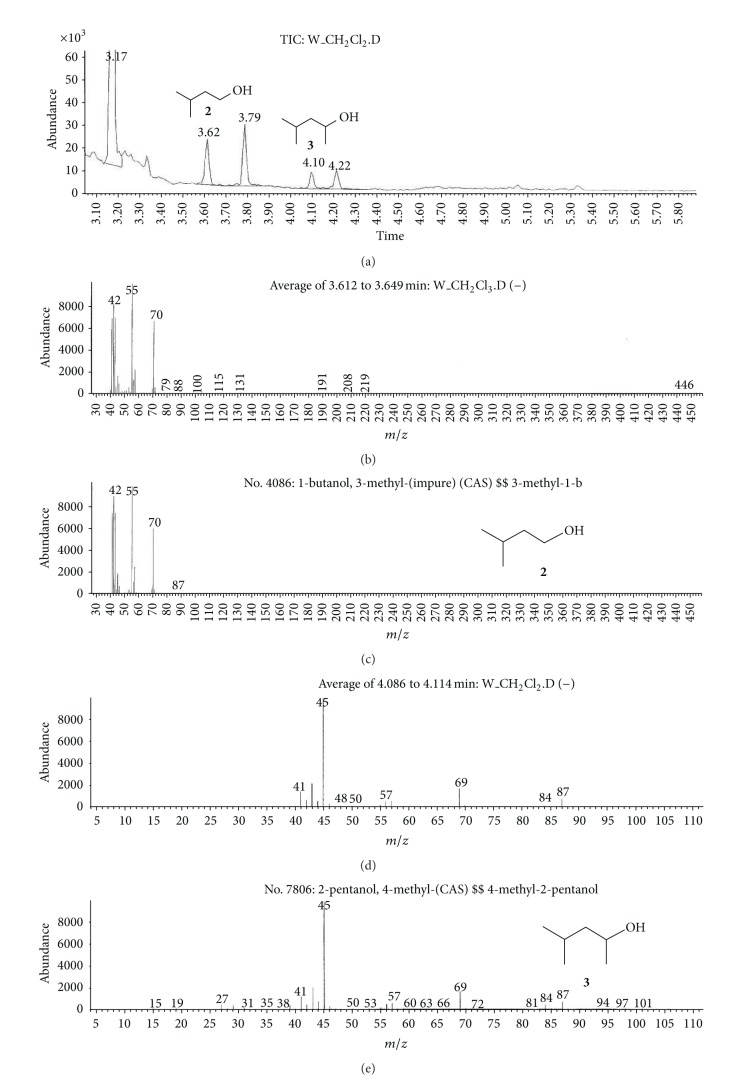
3-Methylbutanol **2** and 1,3-dimethylbutanol **3**, alcohols found in the water dichloromethane extract.

**Figure 3:**
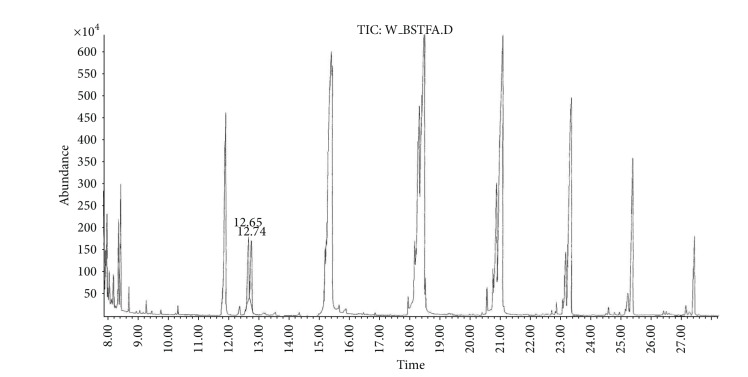
TIC of the water sample silylated with BSTFA showing presence of TMS derivatives of two spiking chemicals at *R*_*T*_ = 12.65 and 12.74 min.

**Figure 4:**
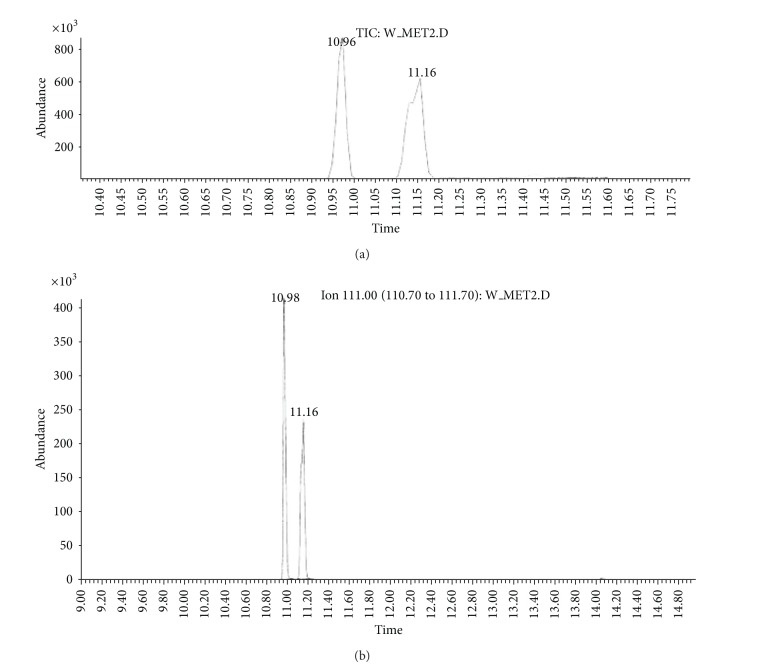
(a) TIC and (b) extracted ion chromatogram at *m/z* 111 of the methylated water sample showing presence of methyl derivatives of two spiking chemicals at *R*_*T*_ = 10.98 and 11.16 min.

**Figure 5:**
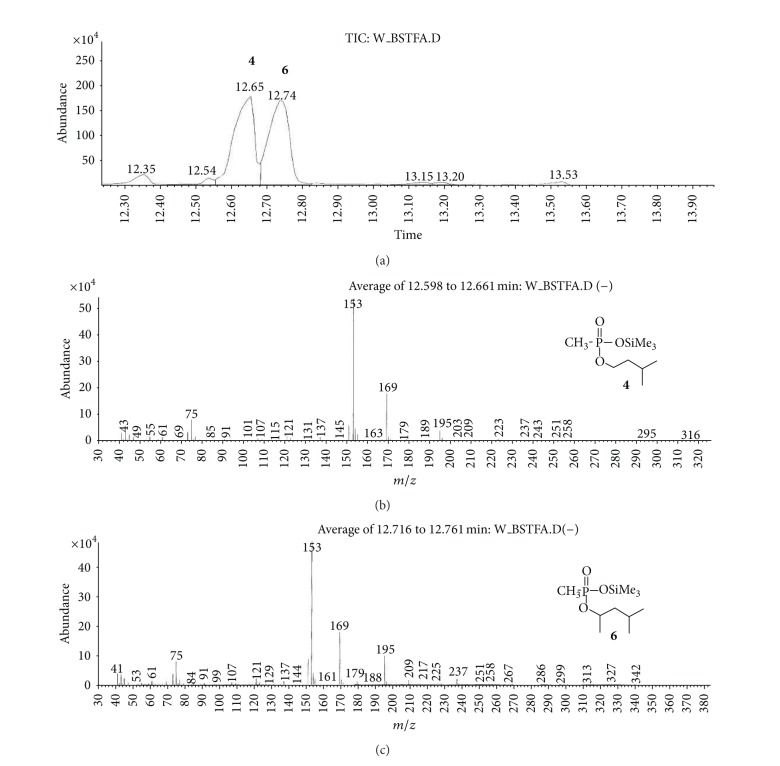
Mass spectra of the TMS derivatives of the spiked chemicals (**I** and **II**) in the water sample. They were identified as *O*-trimethylsilyl-*O*-3-methylbutyl methylphosphonate **4** (silylated **I**) and *O*-trimethylsilyl-*O*-1,3-dimethylbutyl methylphosphonate **6** (silylated **II**).

**Figure 6:**
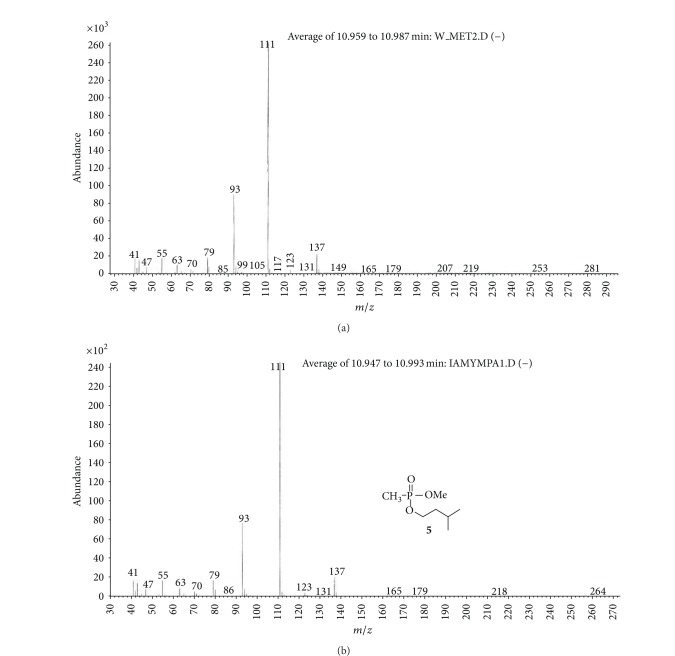
Mass spectrum of Me derivative of spiking chemical **I** in the (W) sample (methylated **I**) (a) was compared with the MS of synthesized reference material—*O*-methyl-*O*-3-methylbutyl methylphosphonate **5** (b). Both MS and *R*_*T*_ were identical.

**Figure 7:**
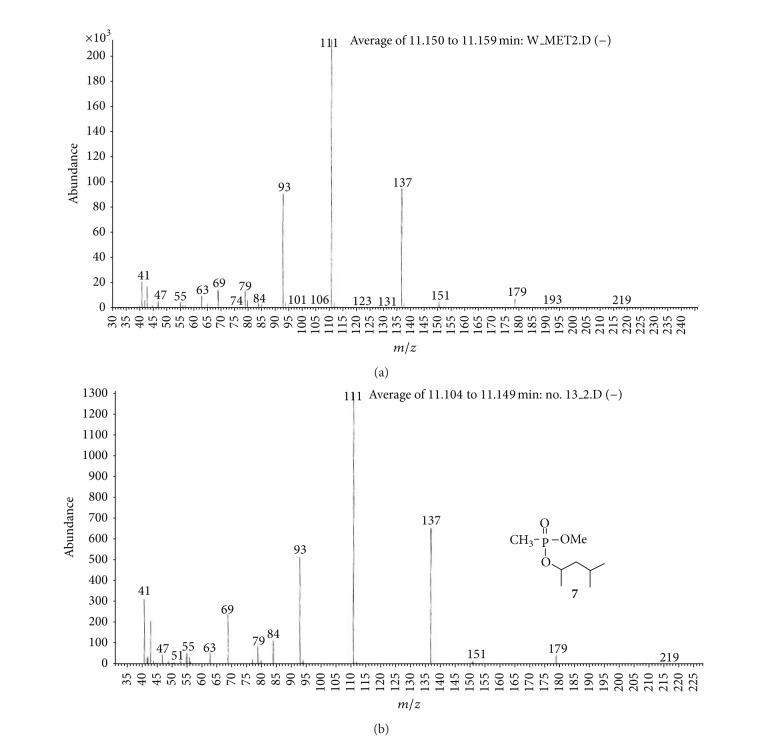
MS of methyl derivative of spiking chemical **II** in the water sample (methylated **II**) (a) was compared with the spectrum of synthesized reference material—*O*-methyl *O*-1,3-dimethylbutyl methylphosphonate **7** (b). Both MS and *R*_*T*_ were identical.

**Figure 8:**
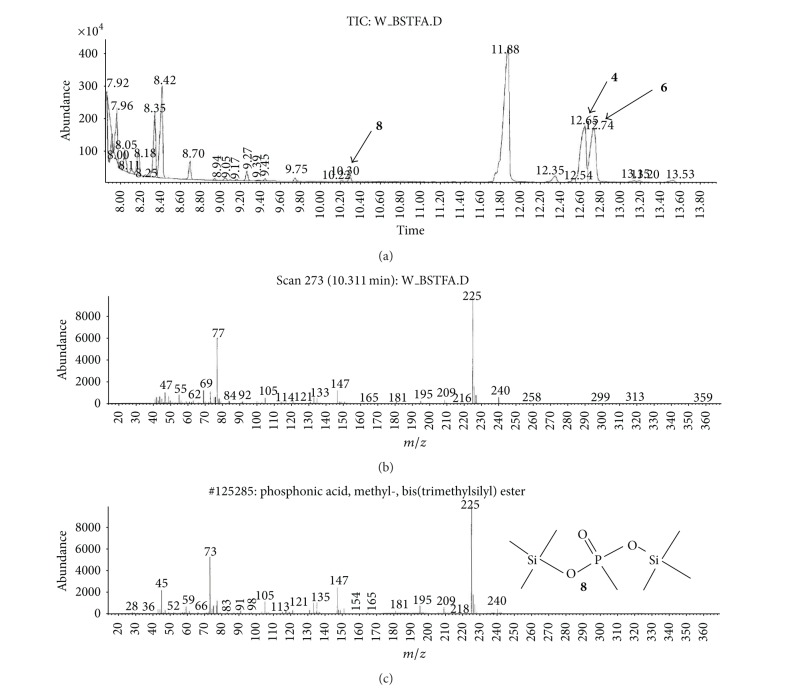
Bis(trimethylsilyl) methylphosphonate **8**, an artifact (a false positive) was formed in the injector by the decomposition of alkyl methylphosphonates.

**Figure 9:**
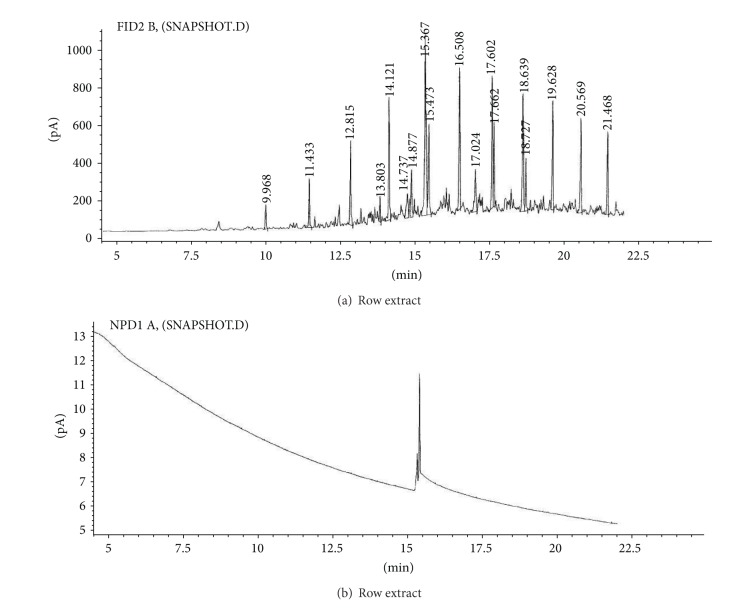
(a) GC/FID and (b) GC/NPD of the raw dichloromethane extract from the soil sample S.

**Figure 10:**
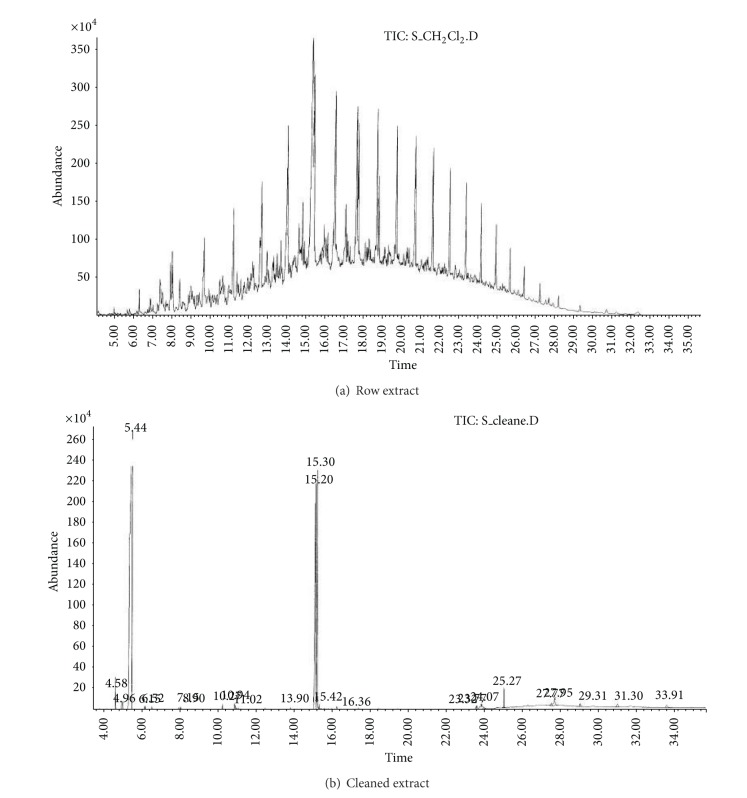
(a) El GC/MS TIC of the raw and (b) cleaned dichloromethane soil extracts.

**Figure 11:**
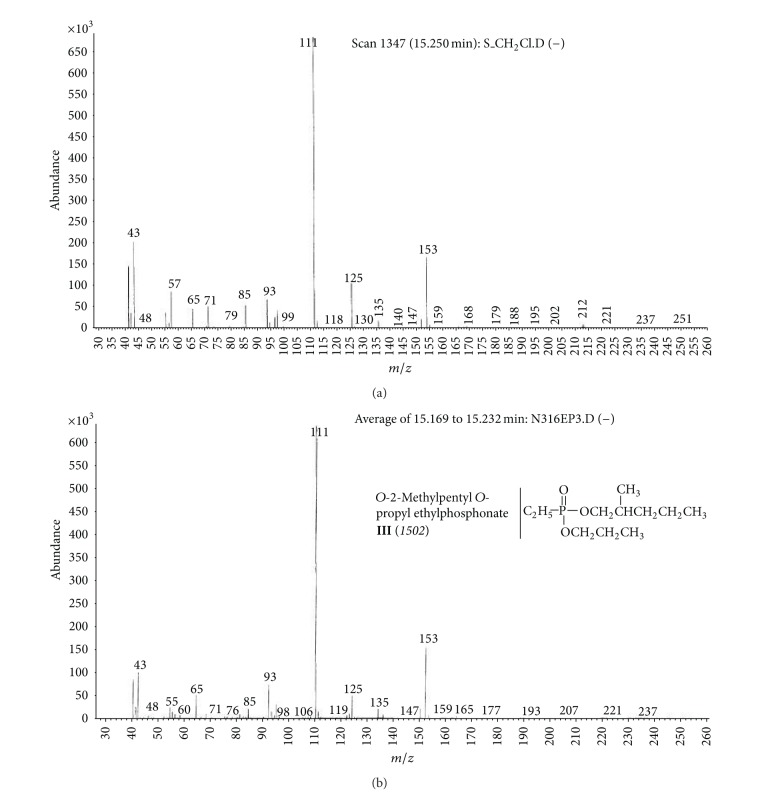
(a) Mass spectra of compound **III** present in the soil sample and (b) of synthesized *O*-2-methylpentyl-*O*-propyl ethylphosphonate **III**.

**Figure 12:**
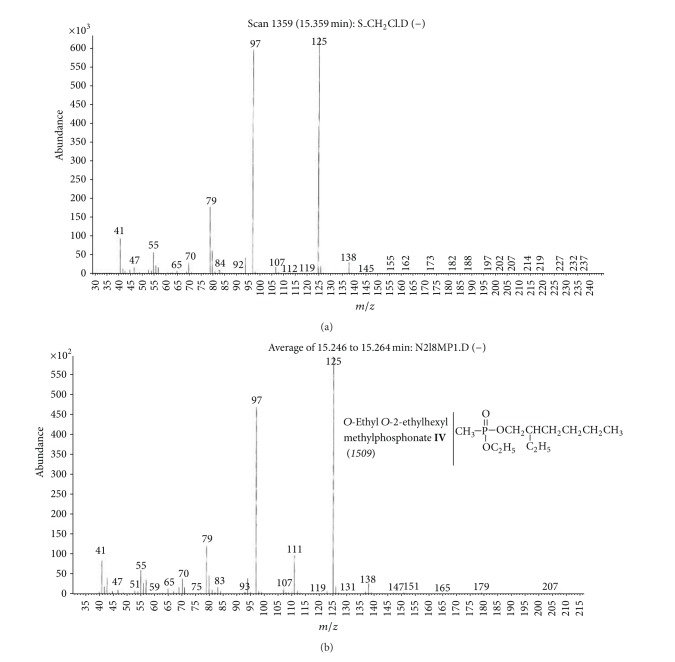
(a) Mass spectra of compound **IV** present in the soil sample and (b) of synthesized *O*-ethyl-*O*-2-ethylhexyl methylphosphonate **IV**.

**Figure 13:**
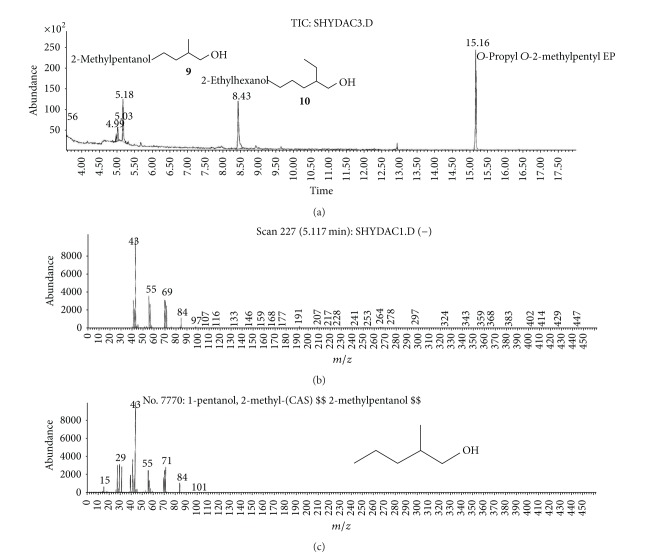
2-Methylpentanol **9** and 2-ethylhexanol **10**, alcohols found in the organic fraction after hydrolysis of the cleaned dichloromethane extract from soil S.

**Figure 14:**
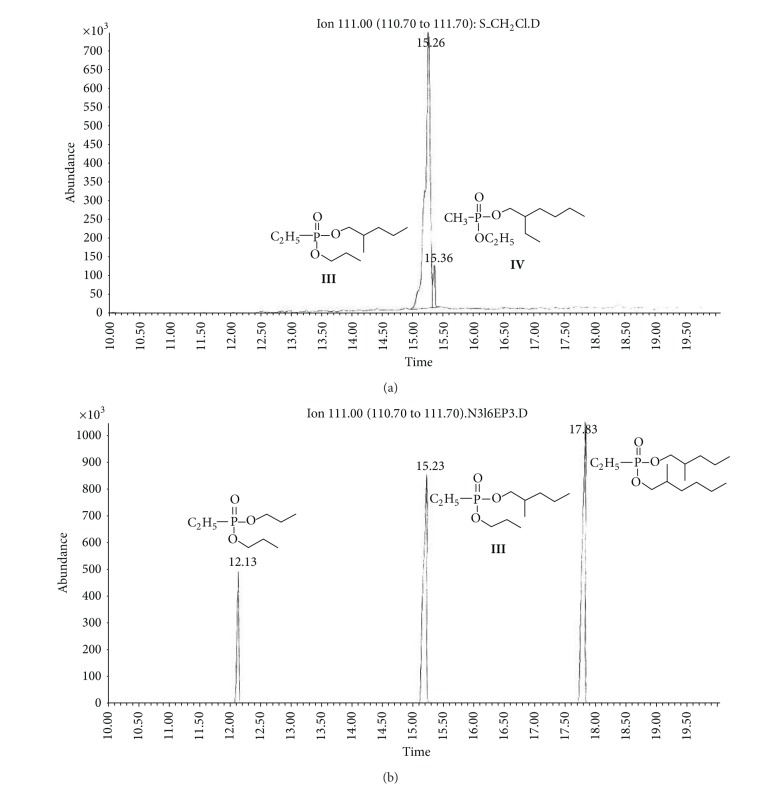
(a) Extracted ion *m/z* 111 chromatogram of compounds **III** (*R*_*T*_ = 15.23 min) and **IV** (*R*_*T*_ = 15.36 min) in the soil sample. (b) Extracted ion *m/z* 111 chromatogram of synthesized *O*-2-methylpentyl-*O*-propyl ethylphosphonate **III** (*R*_*T*_ = 15.23 min), containing two symmetrical analogues: dipropyl ethylphosphonate (*R*_*T*_ = 12.13 min) and bis(*O*-2-methylpentyl) ethylphosphonate (*R*_*T*_ = 17.83 min).

**Figure 15:**
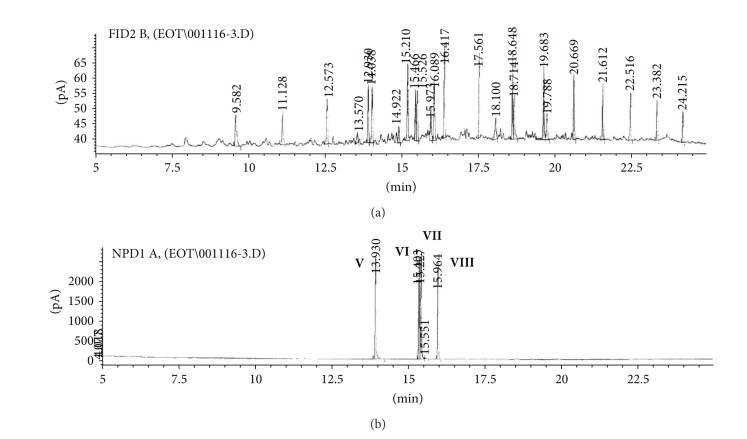
(a) GC/FID and (b) GC/NPD chromatograms of the OE sample comparison showing four N and/or P containing compounds (marked **V**, **VI**, **VII**, and **VIII**).

**Figure 16:**
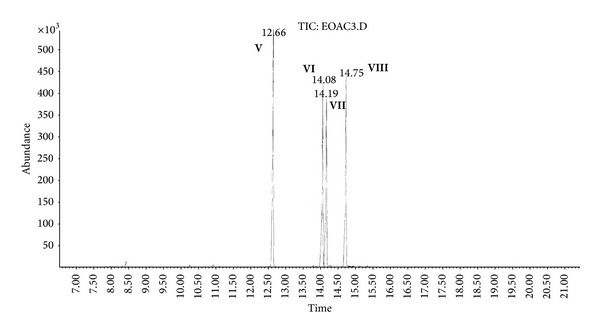
TIC of OE sample: the row sample strongly contaminated with matrix of different organic compounds after cleaning by elution through a cartridge filled with silica.

**Figure 17:**
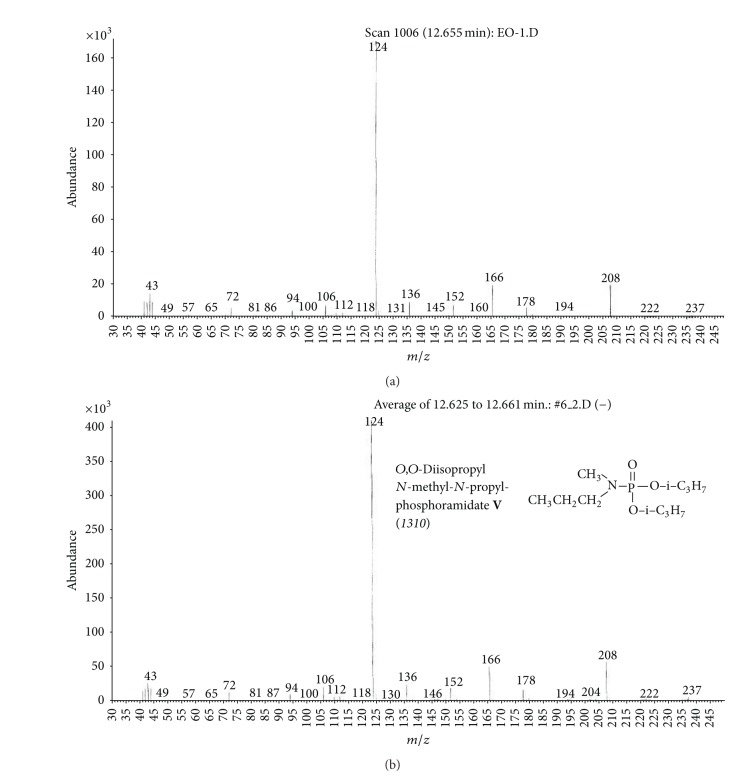
(a) Mass spectrum of compound **V** from the OE sample and (b) MS of *O,O*-diisopropyl *N*-methyl-*N*′-propylphosphoramidate **V**, the synthesized reference standard.

**Figure 18:**
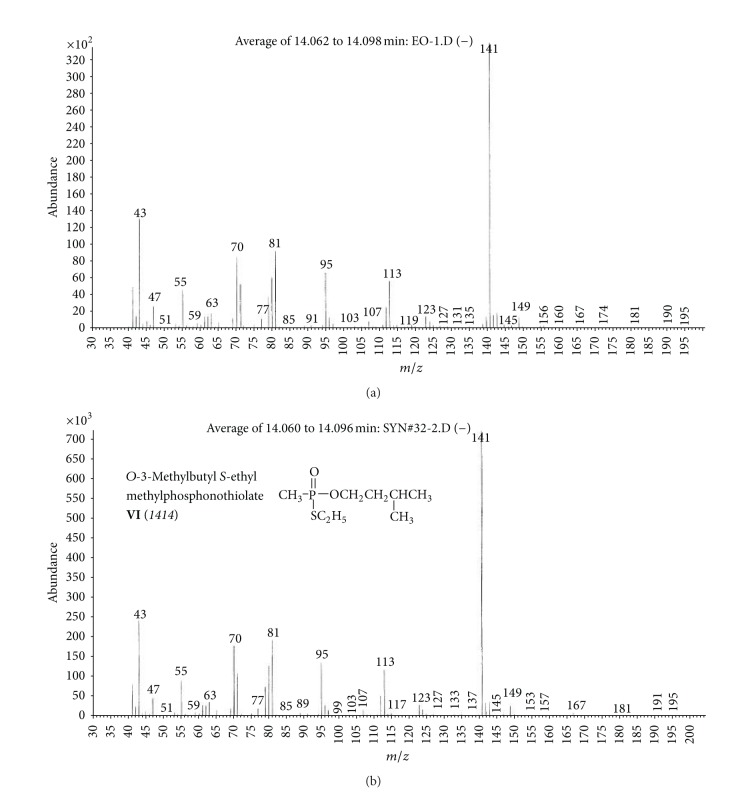
(a) Mass spectrum of compound **VI **from the OE sample and (b) MS of *O*-3-methylbutyl-S-ethyl methylphosphonothiolate **VI**, the synthesized reference chemical.

**Figure 19:**
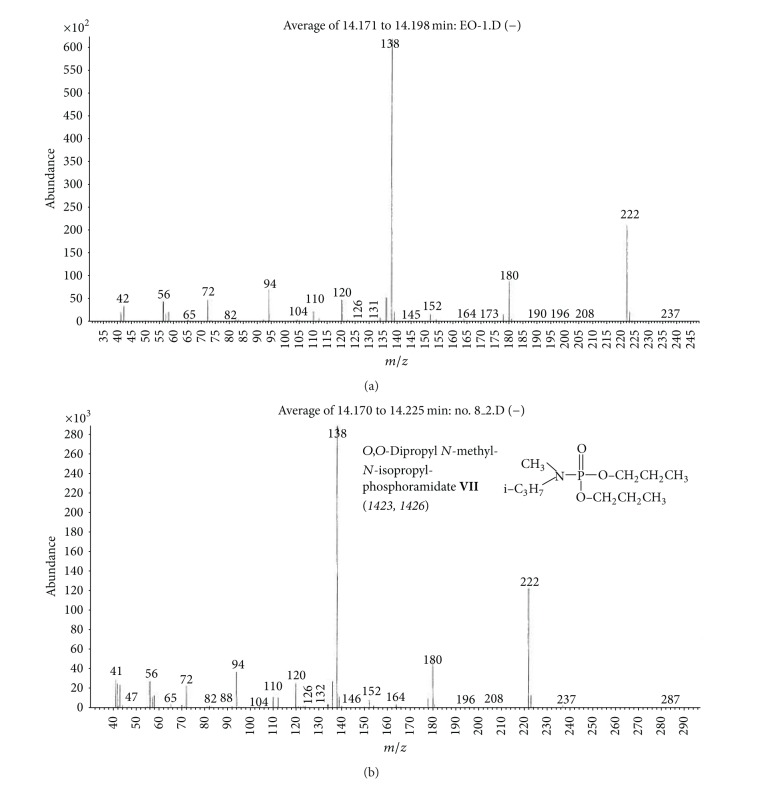
(a) Mass spectrum of compound **VII** from the organic extract sample and (b) MS of *O,O*-dipropyl *N*-methyl-*N*′-isopropylphosphoramidate **VII**, the synthesized reference chemical.

**Figure 20:**
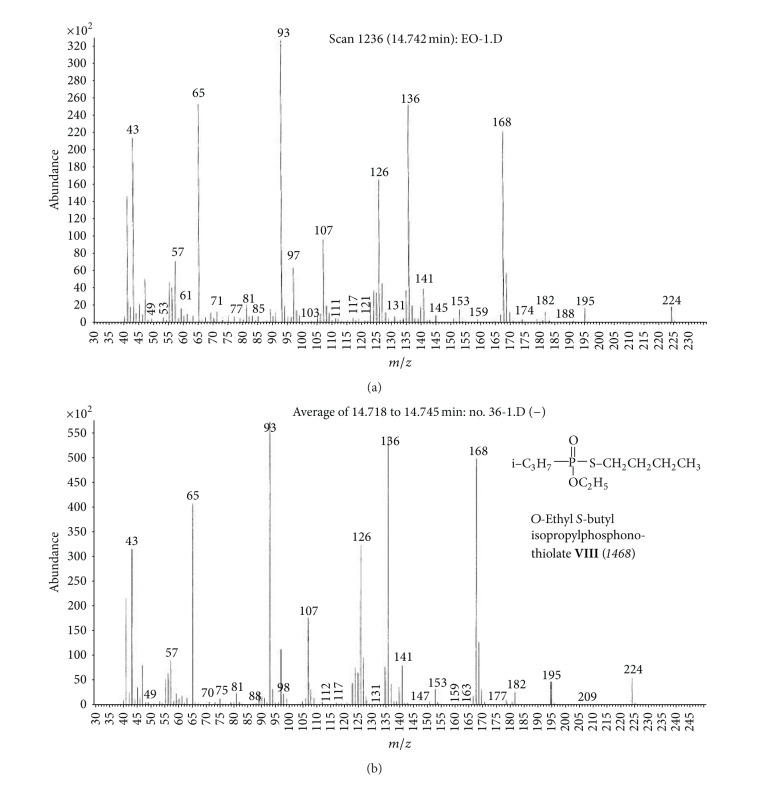
(a) Mass spectrum of compound **VIII** from the organic extract sample and (b) MS of *O*-ethyl-S-butyl isopropylphosphonothiolate **VIII**, the synthesized reference chemical.

**Figure 21:**
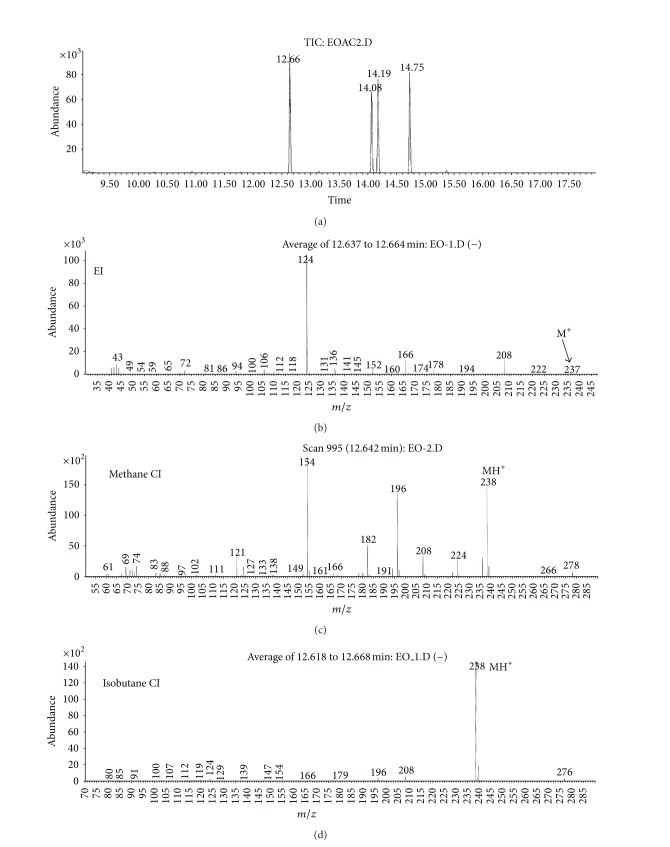
Comparison of electron ionization (El), as well as CH_4_CI and isobutane chemical ionization (CI) mass spectra of *O,O*-diisopropyl-*N*-methyl-*N*-propylphosphoramidate, the compound **V** (*R*_*T*_ = 12.66 min) from the organic extract sample EO.

(a) Water sample (W).

1. First, preliminary results were obtained using GC with NPD/FID detectors. In water sample, practically no major peak which contained phosphorus and nitrogen was observed in the chromatogram of the dichloromethane extract. However, it does not exclude the presence of the sulfur containing compounds, such as HD, its precursors, and analogues, as well as arsenic containing compounds. In the water phase, after evaporation and derivatization with diazomethane and BSTFA, two peaks of appropriate methyl (Me) or trimethylsilyl (TMS) derivatives of two sulfur containing compounds were found.
2. Heavy matrix of polyethylene glycols found in the sample was successfully removed. The polyethylene glycols reextraction to the water phase after derivatization improved the quality of TIC.
3. GC/MS results showed that basically only two scheduled spiking chemicals, nonextractable with dichloromethane but visible after derivatization with diazomethane or BSTFA, were present in the water sample. The compounds were assigned as **I** and **II**. Since they were polar monoalkyl esters of alkylphosphonic acids, they could be identified only as Me and TMS esters.
4. Some observations helped to identify compounds **I** and **II**. Thus, the phosphorus contained compound found in minor quantities in the CH_2_Cl_2_ extract was identified as bis (*O*-3-methylbutyl) methylphosphonate **1** (Figure 1). That may be due to content of an impurity of one of analytes. It shows that this analyte could be a monoester, *O*-3-methylbutyl methylphosphonate **I** (molecular mass l66 u) which was contaminated with its diester analogue **1** (molecular mass 236 u). It was additionally confirmed by the presence of 3-methylbutanol **2** in the same dichloromethane extract (Figure 2).
5. Another alcohol found in the water dichloromethane extract was 1,3-dimethylbutanol **3** (Figure 2). It may indicate that the second spiking chemical in the water was *O*-1,3-dimethylbutyl methylphosphonate **II**, molecular mass 180 u.
6. Further GC/MS data: TIC (Figures 1(a) and 1(b) and Figure 3), extracted ion chromatograms (Figure 4), and mass spectra (Figures 5, 6, and 7) were analyzed, and structure of chemicals **I** and **II** were confirmed in spectra of four derivatives: *O*-trimethylsilyl-*O*-3-methylbutyl methylphosphonate **4** (silylated **I**), *O*-methyl-*O*-3-methylbutyl methylphosphonate **5** (methylated **I**), *O*-trimethylsilyl-*O*-1,3-dimethylbutyl methylphosphonate **6** (silylated **II**), and *O*-methyl-*O*-1,3-dimethylbutyl methylphosphonate **7** (methylated **II**).
7. Very important were not only studying the fragmentation of the mass spectra (which were not all present in the available libraries and databases), but also comparison of the retention indices with the RI database. Particullarly, AMDIS software oriented to scheduled compounds was very useful to find even minor contents of compounds relevant to the CWC among huge peaks of matrix. A paper concerning GC and GC/MS of scheduled compounds was also a very important help [13]. Careful analysis of retention indices permits to eliminate many compounds with similar spectra but definitely different RI.
8. The peaks in the TIC are sometimes split since tetravalent phosphorus atom is asymmetric and if one (or more) carbon atoms are also asymmetric, two (or a power of two) peaks of diastereoisomers with different RI but the same or almost the same mass spectra are observed (soman is a typical example). However, sometimes the “splitting” is only illusory, and in reality there are two different compounds with close RI and similar mass spectra. It was the case of MS of several pairs of compounds in this test, for example, **4** and **6** or **5** and **7**.
9. The bis (trimethylsilyl) ester of methylphosphonic acid **8** was also found in the TIC (Figure 8) and its spectrum, RI being consistent with the literature as well. However, it was found that false positive formed in the injector by the decomposition of alkyl methylphosphonates. To avoid the column was contaminated with scheduled silylated compounds and to avoid false positive identifications, BSTFA was periodically injected into the column to show the contamination degree and to clean the injector from free phosphonic acid residues which could be present in the samples or were produced in the injector.
10. Moreover, the reference substances **I** and **II** were synthesized to finally show that their RI and spectra were identical as the ones found in the water sample.
11. Finally, the methane and isobutane CI GC/MS analyses were performed to show that quasimolecular MH^+^ ion peaks of both derivatized spiked chemicals **I** and **II** corresponded, appropriately, their molecular masses. In CI with isobutane, only the MH^+^ peaks were practically observed in the spectra (soft ionization), whereas, in the methane CI, some fragmentation pathways were visible in addition to a prominent (often 100%) quasimolecular MH^+^ ions or some heavier cationated molecule, for example, MEt^+^ or MC_3_H_5_^+^.
12. Thus, the structures of spiked chemicals were found *O*-3-methylbutyl methylphosphonate, **I** and *O*-1,3-dimethylbutyl methylphosphonate **II**.

(b) Soil sample (S).

1. Preliminary results using the GC/NPD showed that in the soil sample only two compounds containing nitrogen and phosphorus were present, and both were found in the dichloromethane extract from the soil sample S (Figure 9). Neither Me nor TMS derivatives were obtained from any soil extracts.
2. Sample was strongly contaminated with fuel oil and the cleaning procedures needed to be carried out as described in previous sections. EI GC/MS TIC of row (Figure 10(a)) and cleaned (Figure 10(b)) dichloromethane soil extracts were spectacularly different.
3. Preliminary analyses of spectra of these compounds (marked **III** and **IV**) showed both were dialkyl alkylphosphonates with molecular mass 236 u and their spectra were not present in any available MS libraries (TIC: Figure 10 and MSs: Figures 11(a) and 12(a)).
4. Thus, the hydrolysis of the cleaned dichloromethane extract was carried out in a manner described above, and the appropriate alcohols were found in the organic fraction: 2-methylpentanol **9** and 2-ethylhexanol **10** (Figure 13). It should be anticipated that the two other alcohols which composed dialkyl phosphonates **II** and **IV** were too volatile to be identified on GC/MS due to the solvent delay. Derivatization of the resulting free acids remaining after hydrolysis in aqueous fraction with diazomethane and BSTFA afforded dimethyl methylphosphonate **11 **and diethyl methylphosphonate **12**, as well as bis(trimethyl)silyl methylphosphonate **8**, and bis(trimethylsilyl) methylphosphonate **13**, appropriately. That showed that the spiked chemicals **III** and **IV** were dialkyl methylphosphonate and dialkyl ethylphosphonate.
5. Analysis of the spectra and RI permitted determining the structures of these compounds as *O*-2-methylpentyl-*O*-propyl ethylphosphonate **III** and *O*-Ethyl *O*-2-ethylhexyl methylphosphonate **IV**, appropriately.
6. The above was confirmed by (a) synthesis of appropriate compounds and compared the spectra and RI (Figures 11(b)–12(b) and Figure 14) and (b) methane and isobutane CI GC/MS, as it was showed in Figures 4(a) 9 and 10.

(c) Organic extract sample (OE).

1. Preliminary results using the GC/NPD in comparison with GC/FPD showed that, in the OE sample, four N and P containing compounds were present, and they were detected directly in the organic extract. These compounds were marked **V**, **VI**, **VII**, and **VIII**, respectively (Figure 15).
2. Sample was strongly contaminated with matrix of different organic compounds (Figure 15(a)). It needed to be cleaned by chromatographic filtration through a silica bed or flash chromatography using a short column filled with silica and a suitable solvent as a mobile phase (Figure 16(b)).
3. Although the mass spectra of not all detected chemicals were present in the available MS databases, the analyses of spectra fragmentation pathways showed that compounds **V** and **VII** were dialkyl *N,N*′-dialkylphosphoroamidates, both with probable molecular masses 237 u and total number of 10 carbon atoms in all alkyl substituents, but totally different spectra (Figures 17(a)–18(a)). MS of compounds **VI** and **VIII** showed that they were diesters of methyl- and isopropylphosphonic acids containing one atom of sulfur in the molecule. Since the molecular ions ­ at m/z 210 and 224 u, respectively, had very low intensities, it was found that the sulfur was not in the thione (P=S) position, but rather in a thiol (P–S–) structure. Thus, the sulfur containing compounds **VIII** seemed to be *O,S*-dialkyl methylphosphonothiolate (**VI**), with total number of 7 carbon atoms in both alkyl substituents and *O,S*-dialkyl isopropylphosphonothiolate (**VIII**), also with a total number of 7 carbon atoms in all alkyl substituents (Figures 19(a)–20(a)).
4. After chromatographic filtration through a short column, hydrolysis of OE sample was carried out. Unfortunately, the hydrolysis of dialkylphosphoroamidates **V** and **VII** failed. In the water phase, the decomposition products of sulfur that contained compounds were found: butanethiol **14** and diethyl disulfide (C_2_H_5_–S)_2_ **15** (from ethanethiol **16** oxidation), as well as 3-methylbutanol **2** CH_3_–CH(CH_3_)–CH_2_–CH_2_–OH. No phosphonic acid was identified in the water phase. However, these data were sufficient to show that the remaining *O*-alkyl substituent in compound **VIII** should be ethyl.
5. To fully prove structures of spiked chemicals in the organic extract, two series of dialkyl phosphoroamidates and alkylphosphonothiolates were synthesized (Figures 17(b)–20(b)), as well as, finally, methane and isobutane CI GC/MS of all samples were performed (an exemplary comparison of El, CH_4_CI, and isobutane CI mass spectra was showed in Figure 21).
6. Together with other analytical data, the structures of phosphoramidates were found as *O,O*-diisopropyl *N*-methyl-*N*-propylphosphoramidate **V** and *O,O*-dipropyl *N,N*-isopropylphosphoramidate **VII**, whereas sulfur containing compounds was *O*-3-methylbutyl-*S*-ethyl methylphosphonothiolate **VI** and *O*-ethyl-*S*-butyl isopropyl-phosphonothiolate **VIII**.

## 5. Conclusions

**Figure 22:**
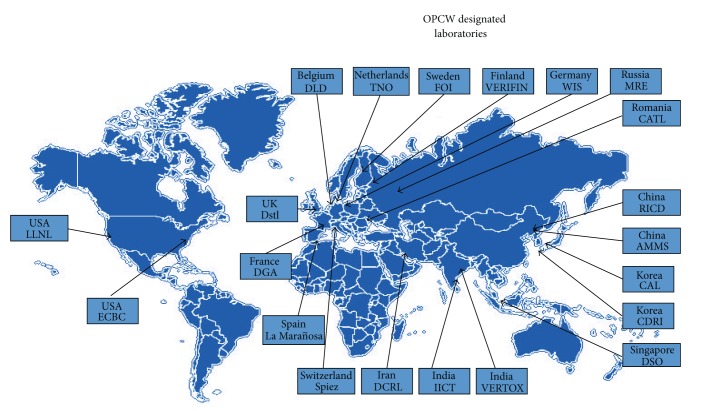
Twenty-one OPCW designated laboratories (as of August 2012) [8].

The presented above example of an interlaboratory proficiency test shows the utility of this method to compare the proficiency of 21 OPCW designated laboratories around the world (Figure 22).

The described above procedures may be used in two special types of OPCW inspections: Challenge Inspection (CI) or Investigation on Alleged Use of Chemical Weapons (IAU). None of them was ever used in practice, but it cannot be excluded that especially the IAU protocols may be used in the next future [14].

Since there are no big differences in chemistry and physicochemical properties between, for example, organophosphorus chemical warfare agents (CWA) and commonly used insecticides, similar methodology and procedures may be also applied for proficiency tests among laboratories looking out for detection, identification, and environmental analyses of various types of chemicals, for example, biologically active compounds, medicines, xenobiotics, pollutants, and so forth. Among them, the important role may play the analytical laboratories working with pesticides and biocides, especially organophosphorus compounds: insecticides, acaricides, or biocides.

## Figures and Tables

**Table 1:** The scheduled chemicals found in the investigated environmental samples.